# Proposals of guidance values for surface contamination by antineoplastic drugs based on long term monitoring in Czech and Slovak hospitals and pharmacies

**DOI:** 10.3389/fpubh.2023.1235496

**Published:** 2023-09-14

**Authors:** Lucie Bláhová, Luěek Bláha, Lenka Doležalová, Jan Kuta, Tereza Hojdarová

**Affiliations:** ^1^RECETOX, Faculty of Science, Masaryk University, Brno, Czechia; ^2^Masaryk Memorial Cancer Institute, Brno, Czechia

**Keywords:** hazardous drugs, surface contamination, antineoplastic drugs, monitoring, technical guidance values

## Abstract

**Introduction:**

The exposures to hazardous antineoplastic drugs (AD) represent serious risks for health care personnel but the exposure limits are not commonly established because of the no-threshold effects (genotoxic action, carcinogenicity) of many ADs. In this study, we discussed and derived practically applicable technical guidance values (TGV) suitable for management of AD risks.

**Methods:**

The long-term monitoring of surface contamination by eight ADs was performed in pharmacies and hospitals in the Czech Republic and Slovak Republic in 2008–2021; in total 2,223 unique samples were collected repeatedly in 48 facilities. AD contamination was studied by LC-MS/MS for cyclophosphamide, ifosfamide, methotrexate, irinotecan, paclitaxel, 5-fluorouracil and gemcitabine and by ICP-MS for total Pt as a marker of platinum-based ADs.

**Results:**

The study highlighted importance of exposure biomarkers like 5-fluorouracil and especially carcinogenic and persistent cyclophosphamide, which should be by default included in monitoring along with other ADs. Highly contaminated spots like interiors of laminar biological safety cabinets represent a specific issue, where monitoring of contamination does not bring much added value, and prevention of staff and separated cleaning procedures should be priority. Rooms and surfaces in health care facilities that should be virtually free of ADs (e.g., offices, kitchenettes, daily rooms) were contaminated with lower frequency and concentrations but any contamination in these areas should be carefully examined.

**Discussion and conclusions:**

For all other working places, i.e., majority of areas in pharmacies and hospitals, where ADs are being prepared, packaged, stored, transported, or administered to patients, the study proposes a generic TGV of 100 pg/cm^2^. The analysis of long-term monitoring data of multiple ADs showed that the exceedance of one TGV can serve as an indicator and trigger for improvement of working practices contributing thus to minimizing of unintended exposures and creating a safe work environment.

## 1. Introduction

A growing number of oncology patients as well as new types of therapy applications (1) leads to increasing use of antineoplastic drugs (ADs). In 2020, more than 19 million new cases of cancer were diagnosed (2). The therapeutic benefits of ADs with carcinogenic, mutagenic, and teratogenic properties outweigh the risks for patients but they represent a risk for health care workers. The long-term occupational exposures have been associated with adverse health outcomes including reproduction toxicity or cancer (3, 4). Acute adverse health effects in such as skin rashes and hair loss have been also reported (5, 6).

Occupational exposures of health care staff to ADs may occur in pharmacies and hospitals through direct dermal contact, inhalation, accidental ingestion, or indirectly via surfaces contaminated by ADs during their preparation, handling or administration to patients (3, 7). To minimize the occupational exposure and achieve maximum product safety, the preparation of ADs is regulated. Preparation of ADs is usually done in laminar or negative pressure boxes (3). In some countries, including Czech Republic, closed systems such as biohazard safety cabinets (BSC) are required by national regulation for AD preparation (8). However, other processes in handling of ADs are often less controlled and may lead to serious occupational exposures of nurses as well as sanitary staff (cleaning of contaminated floors or desktops/tables, handling and washing of contaminated beddings). Recently, exposures to ADs in home care settings have also been documented (9, 10).

The risks of hazardous medicinal products have recently been addressed by authorities around the world. The European Union updated in 2022 the 2004/37/EC Directive on the protection of workers from the risks related to exposure to carcinogens or mutagens at work (Directive (EU) 2022/431),^1^ and a detailed Guidance for the safe management of hazardous medicinal products at work was published in 2023 by the EU Agency for Safety and Health at Work (OSHA).^2^ In parallel, the European Trade Union Institute (ETUI) and the European Biosafety Network (EBN) released the updated list of hazardous medicinal products based on the Regulation (EC) 1272/2008 on the classification, labeling and packaging (CLP).^3^ The ongoing EU Partnership on Risk Assessment of Chemicals PARC (https://www.eu-parc.eu/) also runs the initiative on pan-European evaluation of ADs occupational risks. Also in the USA, the National Institute for Occupational Safety and Health released a detailed document on managing exposures to hazardous drugs in 2023 (11).

While some occupational exposure limit values have been provided in the EU for 58 industrial carcinogens, mutagens and reprotoxic substances (Annex III of EU Directive 2004/37/EC),^4^ no official limits for surface contamination by hazardous medicinal products have been established yet. Correspondingly, national regulations or protocols in health care facilities usually follow the “as low as reasonably achievable” principle (ALARA) to assure low occupational exposures (12). Nevertheless, despite the existing guidelines and prevention measures, monitoring studies still report AD contamination in health care facilities, and the external exposures were confirmed by detection of ADs or their metabolites in urine or blood of health care workers as well as family members of oncology patients (13–18).

Monitoring of contamination in workplaces is an important tool in risk management of these hazardous compounds (3, 10, 12, 19–22), and the standardized wipe sampling of the surfaces is the most broadly used approach to detect contamination by ADs (3). Monitoring results allow to prioritize hot spots, identify major sources, routes of release of ADs during handling, compare situations among health care facilities and positive results often trigger implementation of remedial, and preventive measures (3).

Nowadays, about 100 chemically diverse ADs with various mechanisms of action are used in cancer chemotherapy (3). The consumption of different ADs differ, and some compounds are highly relevant exposure markers with respect to their use and properties such as environmental persistence despite of cleaning procedures (23). For example, in the Czech Republic, about eight ADs are applied intra venously in large quantities including cyclophosphamide (CP), platinum-based drugs (Pt), 5-fluorouracil (FU), paclitaxel (PX), gemcitabine (GEM), irinotecan (IRI), ifosfamide (IF) and methotrexate (MET). These 8 ADs form 50% or more of the AD applications prepared in individual hospitals (9, 10). In agreement with other studies, this shows high importance of few ADs, namely CP, FU and Pt-based drugs as representative markers of occupational exposures (3, 15, 23–25).

The exposure levels in different pharmacy and hospital places may differ by orders of magnitude reaching up to hundreds ng/cm^2^ (documented e.g., for CP and FU) or tens ng/cm^2^ (Pt-based drugs) (15, 25–27). Most commonly, ADs are analyzed in wipe samples from the floors, desktops or various handles (26). Some sites, such as interior of laminar flow boxes are naturally highly contaminated due to open handling of ADs, lower levels are being found at other sites such as storage rooms, outpatient clinics etc. (21, 25, 28–34). On the other hand, these areas, where the procedures and staff are much less controlled represent higher risk to health care workers, e.g., via transdermal absorption (35, 36).

As mentioned above, individual occupational exposure limits for AD in work environment are not commonly established because of the “no-threshold effects” (genotoxic action of many ADs) and poorly understood links with adverse health effects in workers (3, 32). However, for practical reasons, risk managers seek for recommendations such as threshold guidance values (TGV) or hygiene guidance value (HGV). These have been proposed by some authors based on long-term monitoring data sets, e.g., as the 75th, 90th or 95th percentiles of the detected contamination (27, 30, 31, 34, 37, 38). Exceedance of TGVs (or HGVs) indicates that the exposures are not properly controlled and may trigger implementation of measures. Alternatively, a Dutch study (39) suggested a “traffic-light” model for CP considering correlations between the CP levels in the urine of healthcare workers and corresponding surface contamination. This study suggested that surface concentrations of CP < 0.1 ng/cm^2^ might be considered relatively safe (“green”), while CP values above 10 ng/cm^2^ are not acceptable and calls for immediate action (39). Numerically similar guidance values 0.1 ng/cm^2^ for CP and other ADs were suggested further by Connor et al. (20), Kiffmeyer et al. (31), Crul and Simons-Sanders (40), and Korczowska et al. (32) and this value was also highlighted in a document from the European Biosafety Network commenting on amendments of Directive 2004/37/EC on the protection of workers from the risks related to exposure to carcinogens or mutagens.^5^

Based on these evidences, national organizations continue to release recommendations for handling of hazardous drugs in health care sector (3) but debates on guidance values are still open and other important factors such as combined exposures to ADs mixtures remain to be addressed.

The aim of the present study was to exploit a long-term monitoring data of AD contamination in pharmacies and hospitals in the Czech Republic and Slovakia to propose and discuss practically applicable technical guidance values (TGV). Our research shows that different TGVs may be relevant for different specific areas and places within health care facilities, and we discuss three categories. First, the strongly controlled areas where ADs are prepared (AD preparatory rooms). Second, other places in hospitals and pharmacies, where basic personal protective equipment is used such as storage, transport, administration to patients. Third, places expected to be without major contamination such as offices, daily rooms or kitchenettes. The TGVs derived in the present study support evidence-based and tailored risk management as well as benchmarking of surface AD contamination.

## 2. Materials and methods

### 2.1. Material

Analytical standards and solvents were obtained from Toronto Research Chemicals (TRC) or Sigma-Aldrich, British Pharmacopeia Chemical Reference Substances (BPCRS), Analytika (Czech Republic), Merck, and Biosolve BV. More details are provided in Supplementary material. Quality control sample for validation of extraction was prepared in methanol. Field blanks were regularly provided by participating hospitals.

### 2.2. Methods

#### 2.2.1. Design of the monitoring programme

Monitoring program in the Czech Republic runs since 2008 with Slovak Republic added since 2018. It is organized in campaigns two times per year by RECETOX Center at Masaryk University. As of 11/05/2021 (November) the database used for the present paper contained total *N* = 9190 analyses (data points) covering period 2008–2021. This represented *N* = 2,223 unique samples, collected repeatedly in *N* = 48 different pharmacies and/or hospitals. During 2008–2014 only CP and Pt contamination was measured. In 2015, monitoring was further extended with FU, and since 2018–2019 eight ADs (Pt, CP, FU, PX, IF, IRI, GEM, MET) are covered in our monitoring with validated sampling and analytical procedures (21).

Hospitals and pharmacies are invited to voluntarily participate in monitoring, the costs are jointly covered by health care facilities and research grant projects of RECETOX. Participants are provided with standardized sampling kits (described below) and organize own wipe-sampling of surfaces according to the instructions and video manual (https://muni.cz/go/e00d53). Sampling is recommended at the end of a working day or before the next shift, usually before routine daily cleaning in hospitals but the actual sampling strategies reflect needs and decisions of individual participants. The collected wipe-samples are shipped by courier to RECETOX laboratories being responsible for further sample processing, instrumental measurements, and data analyses. The results from each campaign are provided to individual participant, and the participant data are compared with the overall statistics of the annual monitoring. This allows detailed comparing (ranking) of individual hospital/pharmacy within national-wide data. The AD handling procedures at various participants follow generic regulatory recommendations but they cannot be fully harmonized with respect to specific hygiene protocols in different health care providers in Czechia and Slovakia.

#### 2.2.2. Wipe sampling and sample extraction

Surface wipe samples were obtained by standardized procedure (10, 21, 41). Surfaces samples from the pre-marked spots (30 × 30 cm) were obtained with moistened swabs (20 mM acetate buffer, pH 4) and stored at −20°C until extraction. The area of irregular surfaces (such as handles or phones) that could not be marked was calculated after dividing it into regular shapes (e.g., triangles, rectangles, circles) followed by summing up of individual areas. Field blanks (only moistened swab) and quality controls (swab spiked with quality control mixture; CP 3.6 ng/mL, Pt 3.6 ng/mL, FU 7.2 ng/mL, and PX 4.6 ng/mL) were extracted by sonication (45 min; 25 mL of 20 mM acetate buffer pH 4), centrifuged, and the supernatant was used for analyses of organic ADs by LC-MS/MS. For Pt, 0.4 mL aliquot of the supernatant was diluted with 2 ml of 3% hydrochloric acid and analyzed by ICP-MS.

The recoveries of the wipe and extraction procedures from different surfaces were validated in our previous studies (21, 41). Briefly, for CP, Pt, FU mean recovery for all tested surfaces was > 90%. For other monitored compounds - PX, IF, GEM, IRI, MET - mean recoveries were 80, 94, 94, 96, 47%, respectively, for the stainless-steel surface, and 67, 92, 88, 47, 26 %, respectively, for the benchtop material (see Supplementary Table 3 for details).

#### 2.2.3. Instrumental analyses of ADs

Liquid chromatography/tandem mass spectrometry, LC-MS/MS Agilent 1200 coupled with Agilent 6410 Triple-Quad MS was used for analyses of CP between 2008 and 2015 (21). Since 2015, Waters Acquity LC chromatograph (Waters, Manchester, UK) and Xevo TQ-S quadrupole mass spectrometer (Waters, Manchester, UK) were used for multitarget analyses of cyclophosphamide CP, 5-fluorouracil FU, paclitaxel PX, irinotecan IRI, ifosphamide IF, methotrexate MET, and gemcitabine GEM using a recently described multitarget method (41). Analytes were detected in both positive and negative ion modes using tandem mass spectrometry. Settled parameters – i.e., collision energy, cone voltage, retention time as well as the lower limit of quantification, LLOQ, the lowest amount of analyte taken from a known area – 900 cm^2^ - in the sample matrix that can be repeatedly quantified (the signal to noise ratio > 10) are presented in Supplementary Table 1. Data were processed by MassLynxTM software (Waters, Manchester, U.K) and corrected to isotopically labeled standards (CP D4; FU 15N2 13C; PX D5; IRI D10; GEM 13C15N2; MET D3). The results of contamination were reported as picograms of AD per square centimeter of the tested surface (pg/cm^2^).

Inductively coupled plasma/mass spectrometry, ICP-MS used Agilent 7500ce or 7700x ICP-MS systems (Agilent Technologies Inc., Japan) for the analyses of total Pt concentration as a marker of Pt-based ADs (21, 41). Quantification was based on external calibration (194Pt and 195Pt isotopes) with the correction of signal drift and non-spectral interferences on internal standards (185Rh and 209Bi). Results are reported as pg of Pt per square centimeter of surface.

Although the sensitivity of the measurements of long-monitored substances (such as CP and Pt) improved during years because of new instruments, we decided to use the originally derived limits of quantification throughout the present study. This allowed us to assure consistency when comparing frequencies of contamination.

#### 2.2.4. Data analyses

The analyses were done in Microsoft Excel and GraphPad (Boston, MA, USA) and included stratification of original contamination data into categories based on different places of sampling followed by visualization and calculation of basic statistics such as mean, median, min-max, standard deviation, etc.

## 3. Results and discussion

The present study investigated surface contamination in 40 pharmacies (*N* = 1,277 samples) and 43 hospitals (*N* = 946). In addition to this data set, monitoring covered also 17 patient homes (*N* = 133), three retirement houses (*N* = 19), and 2 hospices (*N* = 10) (9) but the data are not considered in the present paper.

From total *N* = 2,223 samples collected in hospitals and pharmacies (field blanks excluded), the most frequently sampled areas were desktops/tables and shelfs (*N* = 1,025) and floors (*N* = 716). Other types of collected samples included interiors of the BSCs, touch displays, handles, fridge doors, outpatient clinic chairs, phones, toilets, etc. The number of yearly AD preparations in participating hospitals varied and hospitals were categorized according to final report of European Commission (42). The monitoring covered small hospital units without own preparation of ADs (*N* = 5), hospitals with low number AD preparations per year (max 5 000; *N* = 17), medium size hospitals with max 15,000 preparation per year (*N* = 8) and large specialized oncology centers (*N* = 18) preparing between 15 000 – 58 000 applications of ADs per year.

The most frequently prepared drugs during 2018–2019 were FU (3 300 preparations per year, median within large specialized oncology hospitals), Pt based drugs (median 2 800 preparations), PX (median 1 004), CP (median 936), GEM (median 732), IRI (median 660), IF and MET (both median of 120 preparations per year) (For detail see Supplementary Table 2).

**Figure 1:**
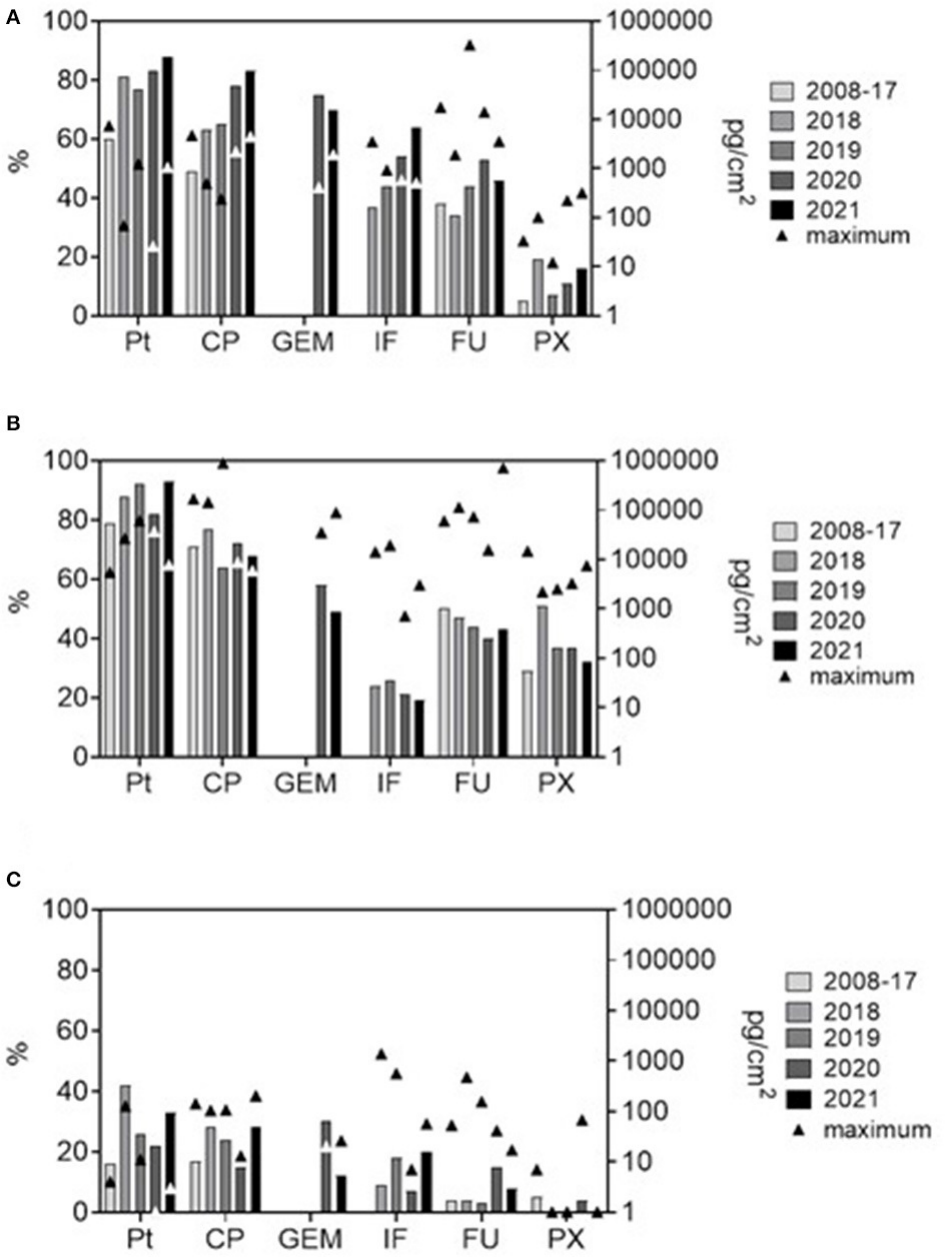
Frequencies of positive samples (%, bars; left Y-axis) and maximum contamination levels (pg/cm^2^, triangle symbols; right Y axis, log scale) for six antineoplastic drugs from the long-term monitoring. **(A)** pharmacies (excluding internal contamination of biological safety cabinets), **(B)** hospital areas (outpatient clinics and patient rooms), and **(C)** offices (offices, staff daily rooms, kitchenettes etc).

Table 1 and Figure 1 show occurrence and contamination by six ADs, i.e., CP and Pt (covering years 2008–2021), FU (2015–2021) and IF, GEM and PX (2018, 2019–2021). The two ADs included in our monitoring - IRI and MET (since 2018) - were only rarely detected with generally low concentrations (Table 1), and they were excluded from follow-up data analyses.

**Table 1:** Contamination of different areas (pharmacies, hospital, offices) and specific sites by six ADs in the Czech Republic.

**Pt**			**N (2008–21)**	**% >LLOQ**	**75th per**.	**90th per**.	**FU**	**N (2015–21)**	**% >LLOQ**	**75th per**.	**90th per**.
	Pharmacy	BSC	74	91%	138	744		88	92%	5 602	19 458
		Work area	486	75%	6	21		352	50%	73	329
		Other	262	53%	2	9		111	19%	< LLOQ	18
	Hospital	WC and outpatient clinic	310	94%	145	679		360	45%	126	596
		Patient and nurse room nurse room	335	77%	13	91		256	46%	143	820
	Office	Office and daily room	133	26%	0.2	1		164	6%	< LLOQ	< LLOQ
**CP**			N (2008–21)	% >LLOQ	75^th^ per.	90^th^ per.	**PX**	N (2016-21)	% >LLOQ	75^th^ per.	90^th^ per.
	Pharmacy	BSC	99	93%	992	3 428		73	53%	35	180
		Work area	600	69%	54	197		287	15%	< LLOQ	7
		Other	321	36%	4	61		88	2%	< LLOQ	< LLOQ
	Hospital	WC and outpatient clinic	400	83%	199	840		315	53%	87	518
		Patient and nurse room	274	53%	12	93		225	17%	< LLOQ	12
	Office	Office and daily room	182	21%	< LLOQ	7		130	2%	< LLOQ	< LLOQ
**GEM**			*N* (2019–21)	% >LLOQ	75th per.	90th per.	**IRI**	N (2018–21)	% >LLOQ	75th per.	90th per.
	Pharmacy	BSC	38	92%	420	1 825		57	65%	60	628
		Work area	125	81%	30	164		226	20%	< LLOQ	8
		Other	37	43%	2	9		74	5%	< LLOQ	< LLOQ
	Hospital	WC and outpatient clinic	121	65%	117	743		275	24%	< LLOQ	33
		Patient and nurse room	78	35%	5	19		198	10%	< LLOQ	< LLOQ
	Office	Office and daily room	52	21%	< LLOQ	3		108	2%	< LLOQ	< LLOQ
**IF**			*N* (2018–21)	% >LLOQ	75th per.	90th per.	**MET**	N (2018–21)	% >LLOQ	75th per.	90th per.
	Pharmacy	BSC	57	75%	104	237		57	23%	< LLOQ	17
		Work area	226	53%	20	84		226	4%	< LLOQ	< LLOQ
		Other	74	46%	6	38		74	1%	< LLOQ	< LLOQ
	Hospital	WC and outpatient clinic	275	13%	< LLOQ	2		275	4%	< LLOQ	< LLOQ
		Patient room and nurse room	198	37%	3	47		198	12%	< LLOQ	3
	Office	Office and daily room	108	14%	< LLOQ	3		108	0%	< LLOQ	< LLOQ

N, number of samples; %>LLOQ, percentage of positive samples; 75th and 90th percentiles, contamination levels in pg/cm^2^. Pt, platinum drugs; CP, cyclophosphamide; GEM, gemcitabine; IF, ifosfamid; FU, 5-fluorouracil; PX, paclitaxel; IRI, irinotecan; MET, methotrexate.

The data were first categorized by main areas with different working regimes (pharmacies, hospital patient areas, offices), and specific sites within these areas. Figure 1 shows the trends in contamination during the years. In Figure 1, specific sites within an area (i.e., within pharmacy and within hospital) were pooled for simplicity, and the most recent situation is highlighted (data collected during early 2008–2017 years are pooled and compared with individual years 2018, 2019, 2020, and 2021).

As apparent, Table 1 clearly shows that ADs were most frequently detected (and often in high concentrations – see 75th and 90th percentile concentrations in Table 1) on surfaces within interiors of BSCs where ADs are being openly handled and prepared for patients. This is in an agreement with other recent studies, where the highest obtained concentrations (up to 9.27 ng/cm^2^ of FU) in BSCs were reported by Sottani et al. (23). Comparably, in Canadian study, maximum contaminations were observed on the floor in front of the BSC (CP up to 120 ng/cm^2^) (19). BSCs thus may serve as an important source of contamination for other hospital areas. Namely in situations when cleaning staff is not well-trained and may spread the contamination from BSCs (23). However, under standard conditions, BSCs are likely to pose lower occupational risk because they are closed under-pressure systems, which minimizes potential impact on pharmacy staff, which is commonly well educated and uses extensive personal protective equipment. Contamination of BSCs thus represents a separate issue with respect to exposure scenario, and data of BSCs contamination were excluded and not used for further discussions of TGVs in hospitals.

As predicted, the results clearly showed that areas, where AD contamination should be virtually avoided (offices, kitchenettes, daily rooms) were, indeed, generally less contaminated. In the offices and related areas, only about 20% of samples were positive for few ADs such as Pt, CP and GEM (see Table 1).

Nevertheless, the overall frequencies of occurrence (Table 1) in pharmacies (BSCs excluded) and hospitals were comparable for most ADs and showed high detection rates namely for Pt, CP, GEM and FU (with overall more than 50% of samples positive). Percent positivity (i.e., % above LLOQ) is a useful parameter to characterize contamination, namely when LLOQs of the analytes are within the same range (43), which was the case also in the present study (see details on LC-MS/MS method in Supplementary Table 1). High positivity in our monitoring is comparable to another recent study from Italy that showed 44% positives in pharmacies and 59% in patient care units for CP, FU, GEM and Pt (23). Importance of carcinogenic CP as a major indicator of surface contamination is confirmed also in recent studies from Canadian hospitals (19) or France (44).

Importantly, our data showed differing time trends. While apparent declines in % positives over the time were observed in hospitals (Figure 1B), there was an opposite trend of increasing positivity in pharmacies (Figure 1A). Further, there were specific differences between hospitals and pharmacies for PX (higher % positive in hospitals) or IF (more frequently found in pharmacies; Table 1). Although decreasing contamination might be expected with regards to long term recognition of the problem and implementation of remedial measures (19, 28, 32, 39), this is not generally confirmed in all reports. Similarly to the present long-term study, variable and non-systematic trends were also reported for FU contamination in Italian hospitals and pharmacies (23) or for GEM, CP and PX in oncology centers in Canada (43). This variability could be related to complexity of health care services including factors such as workload, cleaning regime, national regulatory requirements, solubility of individual drugs, their metabolization or degradation, etc. (https://ec.europa.eu/social/main.jsp?langId=en&catId=89&newsId=10564&furtherNews=yes&).

As a next step, we analyzed data from 2018–2021 to capture the most recent contamination, and, correspondingly, to derive TGVs reflecting the current situation.

Considering different levels of protection of staff in pharmacies and hospitals, areas were categorized into three groups. First, (i) the AD preparation areas, i.e., the isolated room within a pharmacy, where ADs are being prepared for patients, and staff is well protected (usage of whole body coveralls, goggles, face masks and durable gloves). As described above, data on the inner contamination of BSC were excluded. Second, (ii) other AD handling areas such as delivery and storage areas, dispatch rooms in pharmacies as well as outpatient clinics or patient rooms in hospitals including toilets. Within this second category, certain level of staff protection is usually required and used, typically medical gloves. The third category were (iii) the offices, daily rooms, kitchenettes etc., where workers do not use any protective equipment.

**Figure 2:**
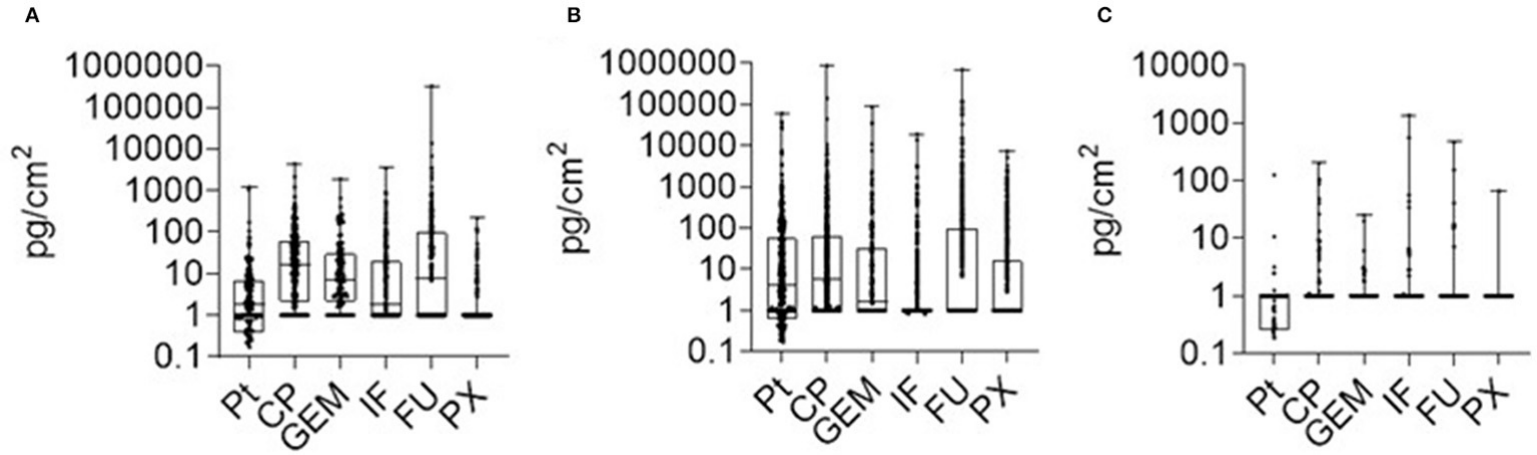
AD contamination (2018–2021) in three categories of areas within health care facilities. **(A)** preparation AD areas (inner parts of BSC excluded); **(B)** other AD handling areas, **(C)** offices and daily rooms. Data show median (line) with 25–75 percentile range (box) and minimum-maximum range.

Figure 2 presents the aggregated 2018–2021 data of contamination, and several generic conclusions could be derived. First, the contamination in areas (i) AD preparation and (ii) other AD handling does not substantially differ, the ranges of contamination for most ADs overlap, the 25^th^-75th quantile range is between 1 and 100 pg/cm^2^. Some specific differences, such as higher PX contamination in hospitals, were discussed above. For the category (iii) offices, contamination was lower with maxima exceptionally exceeding 100 pg/cm^2^. Nevertheless, data revealed AD contamination even in these areas that are used by completely unprotected staff, and periodic monitoring should be recommended to check potential exposures. Any contamination in this category (iii) offices (i.e., surface concentrations above LLOQ) should call for case by case examination and implementation of corresponding measures. Overall, this analysis shows that separate technical guidance values (TGVs) might relevant for different areas corresponding to different exposure scenarios of workers.

With regards to previously derived TGVs, authors used different approaches but a value of 100 pg/cm^2^ (0.1 ng/cm^2^) was repeatedly suggested (20, 31, 32, 39, 40). Table 2 shows the comparison of this threshold with the contamination of (i) AD preparation and (ii) AD handling areas in Czechia and Slovakia. In Table 2, data are additionally categorized to tables and working desktops (i.e., spots commonly touched by hands, i.e., higher risk for workers), and the floors (lower risk of direct contact for most of the workers). The exceedance of 0.1 ng/cm^2^ threshold ranged between 2% of samples from all surfaces (see Pt in category (i) AD preparatory rooms) to 25% exceedance for FU in both (i) AD preparatory and (ii) other AD handling areas. The most frequent exceedances were – in both categories of areas – observed at FU followed by CP and PX. More specifically, within the (i) AD preparatory rooms (upper part of the table), the most contaminated were packaging desktops and transfer carriages (FU and CP followed by GEM and IF). On the contrary, in the (ii) other AD handling areas, threshold was mostly exceeded on the floors, specifically under the administration IV poles in outpatient clinics and around the toilets (FU and CP followed by Pt, GEM and PX).

**Table 2:** Exceedance of 100 pg/cm^2^ threshold originally suggested for CP by Sessink (39) in hospital and pharmacy samples in (i) AD preparation areas, and (ii) other AD handling and drug administration areas.

	**All surfaces**		**Tables**		**Floors**	
	***N***	**%**>**100 pg/cm**^2^	***N***	**%**>**100 pg/cm**^2^	***N***	**%**>**100 pg/cm**^2^
**AD preparation areas**						
Pt	184	2%	128	1%	27	4%
CP	238	15%	168	17%	35	9%
GEM	125	12%	86	14%	17	0%
IF	226	9%	160	11%	32	3%
FU	238	25%	168	25%	35	9%
PX	238	18%	168	18%	35	3%
**Other AD handling areas**						
Pt	446	20%	214	4%	157	42%
CP	553	20%	257	8%	201	36%
GEM	236	18%	102	8%	89	30%
IF	547	5%	254	4%	199	7%
FU	553	25%	257	22%	201	27%
PX	553	15%	257	4%	201	27%

Data from 2018 to 2021 monitoring; samples from biohazard safety cabinets were excluded.

Another derivation of TGVs considers statistical analyses and percentiles based on monitoring data. The exceedance of certain value, such as 90th percentile, indicates that the sample is among the top 10% highest contaminated, which calls for immediate investigation and remedial actions. From the management perspective, a single TGV (75th, 90th or 95th percentile) is another approach and two TGV levels were also discussed in the literature. For example, Schierl et al. (27) reported monitoring of 102 pharmacies in Germany and proposed that contamination of FU and Pt below the 50th percentile indicates a good working practice, while the values higher than 75th percentile called for adaptation of working procedures.

**Figure 3:**
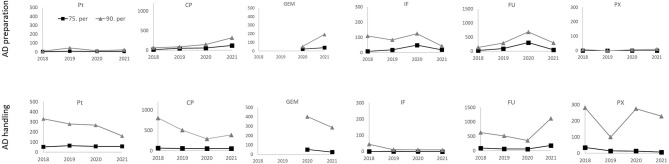
Surface contamination (pg/cm^2^; 75th and 90th percentiles) by 6 antineoplastic drugs from the 2018–2021 monitoring. Results for two categories of areas with differing protection level, i.e., (i) AD preparation areas - upper panels, and (ii) other AD handling areas - lower panels. Results for biological safety cabinets, BSCs, are excluded.

Detailed analysis of percentiles of our monitoring data is shown in Figure 3 and Table 3. The 90th percentile for all ADs was found to be highly variable in different years which is expected for higher percentiles (e.g., compare Figure 3), while the 75th percentile was more stable in time, and, it thus appeared to be more suitable for derivation of a threshold for the workers and their possible exposure to ADs.

**Table 3:** Statistics for surface contamination (pg/cm^2^) by six antineoplastic drugs from the 2018–2021 monitoring.

	**AD preparation areas**					**Other AD handling areas**			
		***N***	**75th per**.	**90th per**.	**95th per**.	***N***	**75th per**.	**90th per**.	**95th per**.
Pt	Tables	128	7	21	26	214	5	26	83
	Floors	27	3	8	17	157	199	747	4 078
	Other	29	8	27	649	75	57	277	830
CP	Tables	168	62	172	262	257	15	69	211
	Floors	35	47	113	170	201	322	976	2 639
	Other	35	58	173	402	95	35	162	1 535
GEM	Tables	86	34	186	241	102	6	60	133
	Floors	17	3	5	7	89	146	690	1 530
	Other	22	53	88	161	45	28	105	542
IF	Tables	160	19	105	209	254	< LLOQ	7	28
	Floors	32	22	49	80	199	2	31	146
	Other	34	14	34	84	94	< LLOQ	67	561
FU	Tables	168	96	350	815	257	63	445	1 072
	Floors	35	20	50	142	201	137	783	1 661
	Other	35	211	477	1 606	95	95	1 870	6 183
PX	Tables	168	< LLOQ	7	16	257	< LLOQ	9	42
	Floors	35	< LLOQ	< LLOQ	18	201	117	592	1 196
	Other	35	< LLOQ	7	12	95	25	478	1 256

Results are shown for two areas with differing protection level, i.e., (i) AD preparation areas, and (ii) other AD handling and drug administration areas and further categorized for Tables, Floors and Other specific spots (door handles, phones, PDA displays, etc).

Detailed statistics (Table 3) show that within the (i) AD preparatory rooms, the 75th percentile was in most cases below the suggested 100 pg/cm^2^. For the second category - (ii) other AD handling areas - contamination of floors was higher with the 75th percentiles exceeding the 100 pg/cm^2^. Desktops/tables and “other” spots (such as door handles) had lower 75th percentiles ranging from <LLOQ to 95 pg/cm^2^ for all six ADs. Similar observations of higher floor contamination with 75th percentiles exceeding the 100 pg/cm^2^ threshold were also reported by other authors such as Hedmer et al. (37) for CP and IF contamination in Sweden or Labrèche et al. (38) for FU in Canada. Similar conclusions were recently published by Dugheri et al. (45) who observed higher floor contamination (compared to desktops), and suggested the new surface exposure level of 100 pg/cm^2^ (with the exception of bathrooms).

Although the floor contamination by ADs is high, direct exposures via skin contact for most of the health care workers is less likely (37). However, this route of exposure is of specific concern for hospital cleaning staff (38), which should be properly trained how to remain protected, and how to avoid spread of ADs from highly contaminated places such as floors or interiors of biosafety cabinets (44). Although decreasing of the floor contamination may be theoretically achievable, e.g., by repeated applications of strong oxidation cleaning products (41), it is challenging and highly demanding considering common hospital practices.

Finally, a potential effort how to better protect health care workers might be using of TGVs that are annually updated based on periodic contamination monitoring. These could further be “tailored” for different places (e.g., floors vs. desktops) or different ADs (AD-specific TGV). Correspondingly, our study suggests that TGVs for floors should be higher than 100 pg/cm^2^ for some ADs so it can be realistically achieved. Such a detailed approach is, however, not very practical for regular hygiene management as many different trigger values might bring uncertainty and confusion. Having one TGV is further supported from our monitoring data, where the 75th percentile for two most important contaminants (i.e., CP and FU) was sufficiently high to serve also as a trigger for other ADs, i.e., Pt, PX, IF and GEM.

## 4. Conclusions

**Figure 4:**
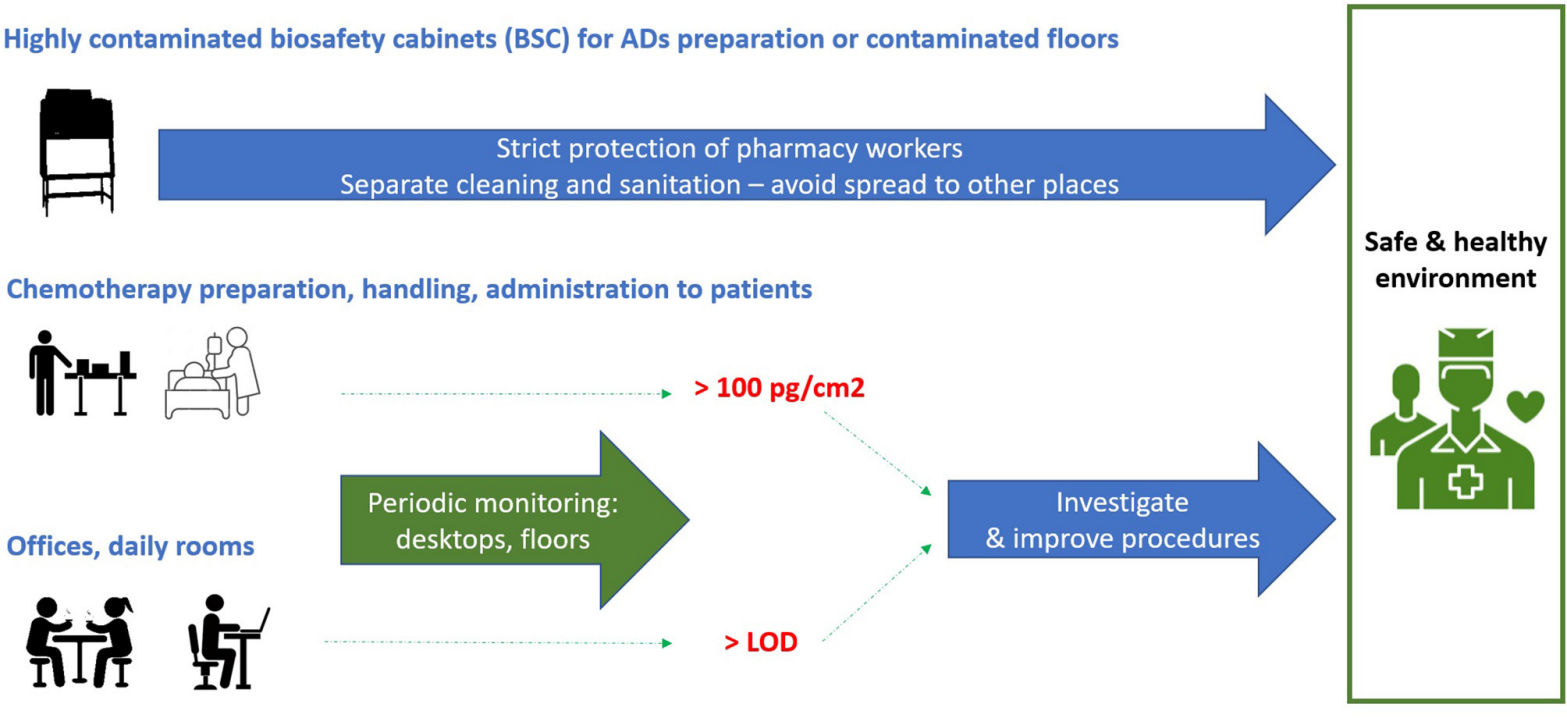
Overview of the main findings and practical recommendations.

The thorough analysis of the long-term monitoring data of AD contamination in Czech and Slovak hospitals revealed following conclusions and recommendations summarized also in Figure 4.

First, it confirmed high relevance of traditional exposure biomarkers such as CP and FU (19). Especially, CP is frequently detected in high concentrations, it is persistent on surfaces (41, 46, 47), and represents a long-term concern considering its carcinogenicity (https://eur-lex.europa.eu/legal-content/EN/TXT/?uri=OJ:L:2022:088:TOC).

Second, highly contaminated spots, namely interiors of laminar biological safety cabinets (BSC) or flow boxes but also contaminated floors represent a major and separate issue. This should be specifically handled by implementing careful cleaning procedures that are separated from other areas preventing thus potential spread of AD contamination. Cleaning and prevention are priority, and monitoring of AD contamination in BSC interiors does not bring much added value, it might be recommended only case by case.

Third, hospital and pharmacy areas that should be virtually free of AD contamination, i.e., offices, kitchenettes, daily rooms, etc., are indeed less contaminated. However, staff is usually not protected in these areas at all, and periodic monitoring should be performed. Any positive contamination by ADs (i.e., samples >LLOQ) should call for immediate examination and adaptation of preventive measures.

Fourth, for the areas in pharmacies and hospitals, where ADs are being prepared, stored, transported and administered to patients, periodic monitoring is needed. A single value of 100 pg/cm^2^ could be suggested as a generic TGV based on the long-term monitoring data of many studies. For most ADs and most exposure situations, this value is close to the 75th percentile (the samples with contamination >100 pg/cm^2^ are among the top 25% contaminated). A TGV of 100 pg/cm^2^ is thus a “warning” or “trigger” value that calls for investigation and improvement of practices, which may be considered during the implementation of new regulations such as the EU Directive 2022/431.

In conclusion, long-life exposures of health care staff to ADs represent a major issue, and routine monitoring along with implementation of proper measures help to implement the “as low as reasonably achievable” principle (ALARA) (12) minimizing thus occupational risks. Challenging problems that require research attention are the take-home anticancer therapies (48), veterinary clinics or research facilities that might contribute to spread of AD contamination to other environments such as patient homes (9).

## Data availability statement

The raw data supporting the conclusions of this article will be made available by the authors, without undue reservation.

## Author contributions

LBlaho contributed to the study design, validated methods, performed analyses of organic drugs, collected data, performed statistical analyses, and drafted the manuscript. JK developed methods, analyzed PT in studied samples, and contributed to manuscript writing. LD contributed to the design of the study, sampling, and manuscript writing. TH contributed to sample extractions, analyses, and to manuscript writing. LBlaho contributed to the study design, method development, data processing, interpretation, and manuscript writing. All authors contributed to the article and approved the submitted version.

## Footnotes

^1^ https://eur-lex.europa.eu/eli/dir/2022/431/oj

^2^ https://osha.europa.eu/en/publications/guidance-safe-management-hazardous-medicinal-products-work

^3^ https://www.europeanbiosafetynetwork.eu/wp-content/uploads/2022/10/The-ETUIs-list-of-hazardous-medicinal-products-HMPs_2022.pdf

^4^ https://echa.europa.eu/carcinogens-mutagens-oels

^5^ https://www.europeanbiosafetynetwork.eu/wp-content/uploads/2019/03/Amendments-to-CMD3-and-implications-1.pdf
