# Supplementary material for: Proposals of guidance values for surface contamination by antineoplastic drugs based on long term monitoring in Czech and Slovak hospitals and pharmacies
Source: Front Public Health. 2023 Sep 14;11:1235496. doi: 10.3389/fpubh.2023.1235496 (PMC10537921; doi:10.3389/fpubh.2023.1235496)
Supplement: Supplementary file 1 [file Table_1.docx]

Supplementary Material

Proposals of guidance values for surface contamination by antineoplastic drugs based on long term monitoring in Czech and Slovak hospitals and pharmacies

# Supplementary Chemicals and reagents

Analytical standards of studied ADs were obtained from Toronto Research Chemicals (TRC) or Sigma-Aldrich: Cyclophosphamide monohydrate, CP (98% purity, TRC); Epirubicin hydrochloride, EPI (97%, TRC); Doxorubicin hydrochloride, DOX (96%, TRC); (+)-Irinotecan, IRI (97%, TRC); Paclitaxel, PX (>96%, Sigma-Aldrich); 5-Fluorouracil, FU (>96%, Sigma-Aldrich); Ifosfamide, IF (98%, TRC); Methotrexate, MET (1mg/mL in methanol with 0.1N NaOH, TRC); Capecitabine, CAP (97%, TRC); Gemcitabine hydrochloride, GEM (95%, TRC), Docetaxel hydrate, DOC (97%, TRC), Cyclophosphamide-d4, CPd4 (98%, TRC); Paclitaxel-d5, PXd5 (99%, TRC); 5-Fluorouracil-13C,15N2, FU13C,15N2 (99%, TRC); Methotrexate-d3, METd3 (99%, TRC); Gemcitabine-13C,15N2 hydrochloride, GEM13C15N2 (95%, TRC), Irinotecan-d10, IRId10 (95%, TRC), Capecitabine-d11, CAPd11 (99%, TRC); Platinum-based ADs were Cisplatin, cisPt (99.5%), Carboplatin, carboPt (99.8%) and Oxaliplatin, oxaliPt (99%) and were purchased from the British Pharmacopoeia Chemical Reference Substances (BPCRS). Stock solutions (1mg/mL) of the standards and internal standards were prepared by dissolving appropriate amounts of substances in methanol or 50% of methanol and stored at -18°C. Positive quality control (QC) sample for validation of extraction and analyses during long term monitoring was prepared in methanol and contained CP (3.6 ng/mL), Pt (3.6 ng/mL; the sum of Pt-drugs), FU (7.2 ng/mL) and PX (4.5 ng/mL). Deionized water was obtained from the Milli-Q Plus system (Millipore, Bedford, MA, USA), formic acid and methanol in UPLC/MS purity were purchased from Biosolve BV, acetic acid (99%), and ammonium acetate from Sigma-Aldrich. Analytical standards for ICP-MS (Pt, Rh, Bi at 1 g/L of each) were purchased from Analytika (Czech Republic). Deionized water (Milli-Q Plus system, Millipore, Bedford, MA, USA) and hydrochloric acid (Suprapur, Merck) were used for the dilution of extracts and stock standards.

# Supplementary Tables

**Supplementary Table 1.** LC-MS/MS parameters of the studied ADs; Limit of quantification (LLOQ);

| compound | retention time | LLOQ | MRM | cone voltage (V) | collision energy (V) |
| --- | --- | --- | --- | --- | --- |
|  | min | pg/cm^2^ | transitions |  |  |
| CP | 7.6 | 1.1 | **261.1 → 106.1** | 36 | 17 |
|  |  |  | 261.1 → 120.1 | 36 | 20 |
|  |  |  | 261.1 → 140.1 | 36 | 24 |
| IF | 7.3 | 1.1 | **261.1 → 92.1** | 40 | 25 |
|  |  |  | 261.1 → 154.1 | 40 | 18 |
| GEM | 2.8 | 1.5 | **264.0 → 112.0** | 25 | 20 |
|  |  |  | 264.0 → 95.0 | 25 | 35 |
| MET | 5.1 | 2.5 | **455.4 → 175.1** | 40 | 40 |
|  |  |  | 455.4 → 308.2 | 40 | 20 |
| IRI | 7.2 | 2.0 | **587.5 → 124.2** | 35 | 30 |
|  |  |  | 587.5 → 167.1 | 35 | 43 |
| PX | 10.3 | 2.7 | **854.4 → 105.0** | 20 | 52 |
|  |  |  | 854.4 → 240.1 | 20 | 30 |
|  |  |  | 854.4 → 286.2 | 20 | 20 |
| FU | 2.1 | 6.9 | **129.0 → 42.0** | 43 | 15 |
|  |  |  | 129.0 → 59.0 | 43 | 22 |
|  |  |  | 129.0 → 86.0 | 43 | 18 |
| CP d4 |  |  | 265.1 → 124.1 | 36 | 24 |
| PX d5 |  |  | 859.6 → 291.2 | 20 | 20 |
| FU 15N2 13C |  |  | 132.3 → 44.3 | 15 | 35 |
| IRI d10 |  |  | 597.3 → 133.2 | 30 | 20 |
| GEM 15N2 13C |  |  | 267.0 → 115.0 | 25 | 15 |
| MET d3 |  |  | 458.3 → 175.0 | 40 | 34 |

**Supplementary Table 2.** Numbers of AD preparations per year (mean 2018-2019) in the 18 Czech Rep. hospitals indicating the use (consumption) of individual drugs. The overall % in the last column shows the relevancy of the ADs covered in the present study that formed a large fraction from all ADs prepared in individual hospitals, na – not available.

| Hospital | All ADs per year | Pt-based drugs | CP | FU | PX | GEM | IRI | IF | MET | % of main ADs preparations |
| --- | --- | --- | --- | --- | --- | --- | --- | --- | --- | --- |
| 1 | 2400 | 180 | 60 | 180 | 216 | 204 | 24 | 0 | 0 | 36 |
| 2 | 3036 | 480 | 156 | 348 | 576 | 240 | 60 | 0 | 12 | 62 |
| 3 | 3600 | 888 | 132 | 1020 | 360 | 240 | 48 | 24 | 12 | 76 |
| 4 | 7200 | 1440 | 180 | 1140 | 480 | 360 | 300 | 0 | 12 | 54 |
| 5 | 7800 | 1680 | 240 | 1680 | 840 | 960 | 840 | na | na | 80 |
| 6 | 16560 | 2724 | 936 | 3060 | 588 | 432 | 444 | 36 | 72 | 50 |
| 7 | 18000 | 1764 | 924 | 3600 | 1044 | 408 | 708 | 96 | 72 | 48 |
| 8 | 20400 | 2040 | 1320 | 1572 | 960 | 648 | 360 | 96 | 204 | 35 |
| 9 | 21600 | 2916 | 624 | 4680 | 1872 | 912 | 708 | 72 | 0 | 55 |
| 10 | 21600 | 3372 | 288 | 2016 | 852 | 336 | 312 | 156 | 120 | 35 |
| 11 | 25200 | 4536 | 1332 | 4836 | 3060 | 1644 | 1092 | 132 | 588 | 68 |
| 12 | 33000 | 3084 | 1440 | 5400 | 2400 | 792 | 636 | 300 | 516 | 44 |
| 13 | 34800 | 4020 | 1200 | 6000 | 1200 | 660 | 1200 | 960 | 1020 | 47 |
| 14 | 42240 | 5100 | 1800 | 9000 | 2952 | 1272 | 864 | 228 | 1512 | 54 |
| 15 | 43200 | 2640 | 1920 | 12000 | 3600 | 1200 | 840 | 240 | 480 | 53 |
| 16 | 45720 | 7044 | 1164 | 11160 | 4884 | 1416 | 1692 | 744 | 84 | 62 |
| 17 | 55512 | 4560 | 3396 | 3360 | 3240 | 1152 | 684 | 108 | 552 | 31 |
| 18 | 57960 | 3072 | 3420 | 3360 | 1440 | 1020 | 840 | 480 | 1128 | 25 |
|  |  |  |  |  |  |  |  | |  |  |

**Supplementary Table 3. Sampling and extraction efficiency of Cyclophosphamide (CP), Ifosfamide (IF), Paclitaxel (PX), Capecitabine (CAP), Irinotecan (IRI), 5-Fluorouracil (FU), Docetaxel (DOC), Gemcitabine (GEM) and Metothrexat (MET)**

| Stainless steel surface | | | | | | | | | | |
| --- | --- | --- | --- | --- | --- | --- | --- | --- | --- | --- |
|  |  | **CP** |  |  | **IF** |  |  | **PX** |  |  |
| Concentration (pg/cm^2^) | 2 | 20 | 200 | 2 | 20 | 200 | 20 | 200 | 2000 |  |
| Extraction recovery (mean, %, N=6) | 100 | 94 | 94 | 96 | 90 | 97 | 87 | 85 | 68 |  |
| Precision (RSD, %, N=6) | 3 | 4 | 3 | 7 | 6 | 5 | 9 | 8 | 11 |  |
|  |  | **CAP** |  |  | **IRI** |  |  | **FU** |  |  |
| Concentration (pg/cm^2^) | 20 | 200 | 2000 | 20 | 200 | 2000 | 50 | 500 | 5000 |  |
| Extraction recovery (mean, %, N=6) | 89 | 89 | 97 | 100 | 92 | 95 | 98 | 89 | 89 |  |
| Precision (RSD, %, N=6) | 7 | 8 | 9 | 12 | 10 | 9 | 11 | 9 | 12 |  |
|  |  | **DOC** |  |  | **GEM** |  |  | **MET** |  |  |
| Concentration (pg/cm^2^) | 20 | 200 | 2000 | 2 | 20 | 200 | 20 | 200 | 2000 |  |
| Extraction recovery (mean, %, N=6) | 99 | 110 | 96 | 101 | 94 | 88 | 53 | 48 | 41 |  |
| Precision (RSD, %, N=6) | 12 | 4 | 3 | 6 | 5 | 7 | 9 | 6 | 10 |  |

| Bench top surface | | | | | | | | | |
| --- | --- | --- | --- | --- | --- | --- | --- | --- | --- |
|  |  | **CP** |  |  | **IF** |  |  | **PX** |  |
| Concentration (pg/cm^2^) | 2 | 20 | 200 | 2 | 20 | 200 | 20 | 200 | 2000 |
| Extraction recovery (mean, %, N=6) | 89 | 94 | 92 | 94 | 90 | 91 | 69 | 68 | 63 |
| Precision (RSD, %, N=6) | 9 | 7 | 7 | 10 | 8 | 4 | 10 | 15 | 10 |
|  |  | **CAP** |  |  | **IRI** |  |  | **FU** |  |
| Concentration (pg/cm^2^) | 20 | 200 | 2000 | 20 | 200 | 2000 | 50 | 500 | 5000 |
| Extraction recovery (mean, %, N=6) | 85 | 108 | 97 | 52 | 43 | 46 | 95 | 84 | 92 |
| Precision (RSD, %, N=6) | 7 | 7 | 5 | 10 | 18 | 7 | 14 | 13 | 10 |
|  |  | **DOC** |  |  | **GEM** |  |  | **MET** |  |
| Concentration (pg/cm^2^) | 20 | 200 | 2000 | 2 | 20 | 200 | 20 | 200 | 2000 |
| Extraction recovery (mean, %, N=6) | 95 | 107 | 89 | 88 | 91 | 86 | 32 | 28 | 18 |
| Precision (RSD, %, N=6) | 7 | 7 | 6 | 6 | 7 | 8 | 4 | 6 | 10 |
